# The optimal neoadjuvant chemotherapy regimen for locally advanced gastric and gastroesophageal junction adenocarcinoma: a systematic review and Bayesian network meta-analysis

**DOI:** 10.1186/s40001-022-00878-7

**Published:** 2022-11-09

**Authors:** Tongya Wang, Changyang Li, Xiang Li, Jing Zhai, Shoulin Wang, Lizong Shen

**Affiliations:** 1 Department of Surgical Oncology, Jiangsu Province Hospital of Chinese Medicine, Affiliated Hospital of Nanjing University of Chinese Medicine, Nanjing, China; 2 Department of General Surgery, Affiliated Hospital of Integrated Chinese and Western Medicine, Nanjing University of Chinese Medicine, Nanjing, China; 3 School of Public Health, Nanjing Medical University, Nanjing, China; 4 Department of General Surgery, The First Affiliated Hospital, Nanjing Medical University, Nanjing, China

**Keywords:** Gastric cancer, Gastroesophageal junction cancer, Neoadjuvant chemotherapy, Systematic review, Bayesian network meta-analysis

## Abstract

**Background:**

Neoadjuvant chemotherapy (NAC) for locally advanced gastric and gastroesophageal junction adenocarcinoma (LAGC) has been recommended in several guidelines. However, there is no global consensus about the optimum of NAC regimens. We aimed to determine the optimal NAC regimen for LAGC.

**Methods:**

A systematic review and Bayesian network meta-analysis was performed. The literature search was conducted from inception to June 2022. The odds ratio (OR) value and 95% confidence interval (95% CI) were used for assessment of R0 resection rate and pathological complete response rate (pCR) as primary outcomes. The hazard ratio (HR) value and 95% CI were interpreted for the assessment of overall survival (OS) and disease-free survival (DFS) as second outcomes. The risk ratio (RR) value and 95% CI were used for safety assessment.

**Results:**

Twelve randomized controlled trials were identified with 3846 eligible participants. The network plots for R0 resectability, OS, and DFS constituted closed loops. The regimens of TPF (taxane and platinum *plus* fluoropyrimidine), ECF (epirubicin and cisplatin *plus* fluorouracil), and PF (platinum *plus* fluoropyrimidine) showed a meaningful improvement of R0 resectability, as well as OS and/or DFS, compared with surgery (including surgery-alone and surgery *plus* postoperative adjuvant chemotherapy). Importantly, among these regimens, TPF regimen showed significant superiority in R0 resection rate (*versus* ECF regimen), OS (*versus* ECF regimen), DFS (*versus* PF and ECF regimens), and pCR (*versus* PF regimen).

**Conclusions:**

The taxane-based triplet regimen of TPF is likely the optimal neoadjuvant chemotherapy regimen for LAGC patients.

**Supplementary Information:**

The online version contains supplementary material available at 10.1186/s40001-022-00878-7.

## Background

Despite many progresses made in early detection of gastric and gastroesophageal junction (GEJ) adenocarcinoma in the past decades, more than two-thirds of patients are diagnosed at advanced stages, and the overall survival remains low [1, 2]. In addition to radical surgery, multimodal approaches for gastric and GEJ cancer have been proposed to improve the prognosis. Preoperative chemotherapy for gastric and GEJ cancer was first reported in 1976 [3]. Until the MAGIC trial was reported in 2006, the perioperative chemotherapy with ECF regimen (epirubicin and cisplatin *plus* fluorouracil) for resectable adenocarcinoma of the stomach has been demonstrated to decrease tumor size and stage, and significantly improve progression-free and overall survival, as compared with surgery-alone [4]. In 2007, ECF regimen was officially recommended in the National Comprehensive Cancer Network (NCCN) guideline as category 1 evidence of neoadjuvant chemotherapy (NAC) for gastric cancer [5]. Since then, an increasing number of clinical trials concerning NAC for gastric and GEJ cancer have been conducted. NAC is better tolerated than postoperative adjuvant chemotherapy [6, 7]. Further, NAC improves the chance of radical resection [8], and eradicates potential micrometastases, and evaluates the chemosensitivity [4, 9]. Thus, NAC has gradually become the preferred approach for localized advanced gastric and GEJ cancer (LAGC). Currently, the guidelines for gastric and GEJ cancer of NCCN, European Society for Medical Oncology (ESMO), Japanese Gastric Cancer Association (JGCA) and Chinese Society of Clinical Oncology (CSCO) have described numerous recommended neoadjuvant treatments [7, 10–12].

However, there is no global consensus about the optimum of NAC regimens for LAGC. In clinical practice, physicians select a certain NAC regimen for specific patient mainly depending on the patient’s performance status in combination with physicians’ experience. Although accumulating meta-analyses have demonstrated significant survival or other benefits in favor of NAC or neoadjuvant chemoradiotherapy *versus* surgery in patients with gastric and GEJ adenocarcinoma [13, 14], there is still a lack of systematic evaluation of effectiveness and safety of each NAC regimen for determining the optimal regimen.

Unlike the conventional meta-analysis, network meta-analysis allows comparison of multiple NAC schemes in which there is no head-to-head comparison [15, 16]. Herein, a systematic review and Bayesian network meta-analysis was performed. Twelve randomized controlled trials (RCTs) concerning NAC for LAGC were identified from 17 published papers [4, 9, 17–31], which involved 11 regimens. To facilitate analysis, these regimens were categorized into four types based on pharmacology, named TP regimen [taxane (*paclitaxel*) *plus* platinum (*cisplatin*)], PF regimen [platinum (*cisplatin* or *oxaliplatin*) *plus* fluoropyrimidine (*Fluorouracil* or *tegafur gimeracil oteracil potassium capsule (S-1)* or *capecitabine*)], ECF regimen (epirubicin *and* cisplatin *plus* fluorouracil/capecitabine), and TPF regimen [taxane (*docetaxel*) *and* platinum (*cisplatin* or *oxaliplatin*) *plus* fluoropyrimidine (*fluorouracil* or *S-1*)]. The results revealed that the regimens of TPF, ECF, and PF showed a clinically meaningful improvement of R0 resectability, as well as overall survival (OS), and/or disease-free survival (DFS), compared with surgery. Importantly, among these regimens, TPF regimen showed significant superiority in R0 resection rate (*versus* ECF regimen), OS (*versus* ECF regimen), DFS (*versus* PF regimen and ECF regimen), and pathological complete response rate (*versus* PF regimen). TPF regimen showed a relatively higher incidence of the grade 3/4 adverse events than others, while the postoperative 30-day mortality in ECF regimen was increased compared those in TPF regimen and PF regimen. Collectively, this study provides a novel and definitive evidence that taxane-based triplet regimen, TPF, is an optimal NAC regimen for LAGC patients with good performance status.

## Methods

This study was in compliance with the Preferred Reporting Items for Systematic Reviews and Meta-Analyses extension statement for network meta-analyses (PRISMA-NMA) [32]. The protocol was registered with PROSPERO (CRD42022303124). The study was performed basing on the information from published literatures, and the Institutional Review Board exemption was granted for the innocuousness of the review study.

### Search strategy

The literature search was conducted using PubMed, Embase, Web of Science, Cochrane Library, and ClinicalTrials.gov published in English from inception to June 2022. The search terms and the detailed retrieval strategies are listed in Additional file 3: Table S1. To avoid omission, the important conference abstracts were included, and the ongoing RCTs were followed for the latest advances, and the relevant references of the included articles were further searched. For the literatures published at different stages of the same study, the data were merged. The bibliographies of included studies were also checked for additional trials.

### Study selection

The inclusion criteria included (1) phase II/III randomized controlled trials (RCTs); (2) histologically primary locally advanced adenocarcinoma of the stomach or gastroesophageal junction (GEJ) with no evidence of distant metastases or other unresectable factors; (3) studies comparing two or more treatment regiments of surgery (including surgery-alone and surgery *plus* postoperative adjuvant chemotherapy) and/or NAC; (4) two or more cycles of NAC, and (5) studies containing at least one efficacy and/or safety outcome, including R0 resection rate, pathological complete response (pCR), OS, DFS, grade 3/4 adverse events, and postoperative 30-day mortality.

The RCTs of radiotherapy, chemoradiotherapy, targeted therapy or immunotherapy, were excluded. The trials involving methotrexate and mitomycin, which were seldom used in gastric cancer chemotherapy currently, were also excluded [33, 34].

### Data collection and study quality assessment

Two investigators independently performed literature searching, reviewing, and information extracting to a pre-determined spreadsheet. Any disagreement was resolved through discussion with another independent reviewer.

If the original literature did not provide the hazard ratio (HR) and 95% confidence interval (95% CI), the Engauge Digitizer and statistical formula were used to extract and calculate the natural logarithm (ln) of HR (ie, ln [HR]) and the standard error (SE) of the ln multiplied by the HR (ie, SE (ln [HR])) [35].

For safety assessment, the numbers of grade 3/4 adverse events and the deaths within 30 days after surgery that occurred in different NAC regimens were collected. The probability of an adverse event in each regimen was expressed as a percentage.

The Cochrane risk-of-bias tool was used to assess the risk of bias in an individual study, which is based on random sequence generation, allocation concealment, blinding of participating personnel, blinding of outcome assessment, outcome data integrity, selective reporting, and other biases. The risk of bias was assessed as high, low, or unclear.

### Quality of evidence

The quality of evidence is assessed using four-step approach according to the Grading of Recommendations Assessment, Development and Evaluation (GRADE) [36], which is mainly based on the study limitations, reporting bias, inconsistency of results, indirectness of evidence and imprecision. The GRADE rates the quality of evidence as high, moderate, low, and very low quality. High quality of evidence represents the strong confidence in the estimate of effect [37].

### Statistical analysis

All direct and indirect evidence were pooled to compare the efficacy and safety of different NAC regimens. The odds ratio (OR) value and 95% CI were used for assessment of R0 resection rate and pCR as primary outcomes. The corresponding HR value and 95% CI were interpreted for assessment of OS and DFS as second outcomes. The risk ratio (RR) value and 95% CI were used for safety assessment.

Statistical models based on the Bayesian framework were constructed using “gemtc” package in R (version 4.1.2) (RStudio, Boston, MA). The network plots were generated using Stata (version 14.0) (StataCorp, Texas, USA). For each analysis, four Markov chains were set, and each chain produced 50,000 iterations with the 20,000 iterations discarded as burn-in period. Convergence of iterations was assessed with trace plots and the Gelman–Rubin–Brooks statistic [38]. The ranking probability of regimens was estimated by calculating the surface under the cumulative ranking curve (SUCRA) for each regimen [39]. The SUCRA value ranges from 0 to 100%, where the regimens with higher SUCRA values are represented to have better efficacy.

To evaluate the consistency of direct and indirect evidence, the model fit was assessed by comparing the consistent model with the inconsistent model, and the node-splitting method was used to explore the local inconsistency [40]. *Q* test and *I*^2^ were used to assess the heterogeneity of studies. *I*^2^ values less than 25% were considered as low heterogeneity, 25–50% as medium heterogeneity, and above 50% as high heterogeneity. A comparative-adjusted funnel plots were used to identify the small sample effect between studies and to test publication bias.

## Results

**Fig. 1:**
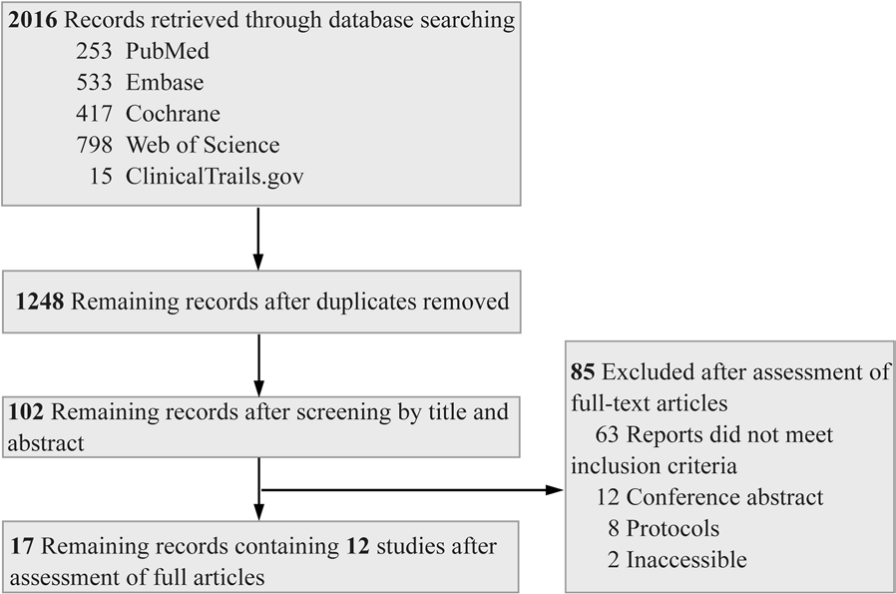
Flowchart of literature retrieval

The literature retrieval process is shown in Fig. 1. Twelve RCTs were included in this study. A total of 3846 eligible participants were recruited, with sample size between 69 and 1022 patients. The characteristics of the included studies are shown in Table 1. As shown in Additional file 4: Table S2, the global *I*^2^ values and the results of local inconsistency test indicated that the fixed-effects models were suitable for analyzing R0 resection rate, OS, and DFS.

**Table 1:** The characteristics of the enrolled clinical trials in this systematic review and network meta-analysis

Authors	Country	Study start and end time	Intervention group			Control group		
			NAC/cycles	Pat	Postoperative/cycles	NAC/cycles	Pat	Postoperative/cycles
Cunningham et al. [4]	UK	1994.7–2002.4	Epirubicin + cisplatin + fluorouracil/3	250	Epirubicin + cisplatin + fluorouracil/3	NA	253	NA
Schuhmacher et al. [9]	Germany	1999.7–2004.2	Cisplatin + fluorouracil/2	72	NA	NA	72	NA
Ychou et al. [17]	France	1995.11–2003.12	Cisplatin + fluorouracil/3	113	Cisplatin + fluorouracil/3	NA	111	NA
Biffi et al. [18]; Fazio et al. [19]	Italy	1999.11–2005.11	Docetaxel + cisplatin + fluorouracil/4	34	NA	NA	35	Docetaxel + cisplatin + fluorouracil/4
Yoshikawa et al. [20, 21]	Japan	2009.10–2011.7	Cisplatin + S-1/2–4	41	S-1 (6–12 m)	Cisplatin + paclitaxel/2–4	42	S-1 (6–12 m)
Xue et al. [22]	China	2011.09–2012.12	Oxaliplatin + S-1/oxaliplatin + capecitabine/2	50	Oxaliplatin + S-1/oxaliplatin + capecitabine/6	NA	50	Oxaliplatin + S-1/oxaliplatin + capecitabine/8
Al-Batran et al. [23, 24]	Germany	2010.8–2015.2	Epirubicin + cisplatin + fluorouracil/capecitabine/3	360	Epirubicin + cisplatin + fluorouracil/capecitabine/3	Docetaxel + oxaliplatin + fluorouracil/4	356	Docetaxel + oxaliplatin + fluorouracil 4
Hayashi et al. [26]	Japan	2011.10–2014.9	Cisplatin + S-1/2–4	62	S-1 (12 m)	Docetaxel + cisplatin + S-1 (2–4)	65	S-1 (12 m)
Sah et al. [27]	China	2018.8–2020.3	Docetaxel + oxaliplatin + fluorouracil/4	40	Docetaxel + oxaliplatin + fluorouracil/NA	Oxaliplatin + S-1/3	34	Oxaliplatin + S-1/NA
Terashima et al. [28]; Iwasaki et al. [29]	Japan	2005.10–2013.7	Cisplatin + S-1/2	151	S-1/8	NA	149	S-1/8
Kang et al. [30]	Korea	2012.1–2017.1	Docetaxel + oxaliplatin + S-1/3	238	S-1/	NA	246	S-1/8
Zhang et al. [31]	China	2012.8–2017.2	Oxaliplatin + S-1/3	337	Oxaliplatin + S-1/5	NA	685	Oxaliplatin + S-1/capecitabine/8

*NAC* neoadjuvant chemotherapy, *Pat* patient number

### R0 resection rate

**Fig. 2:**
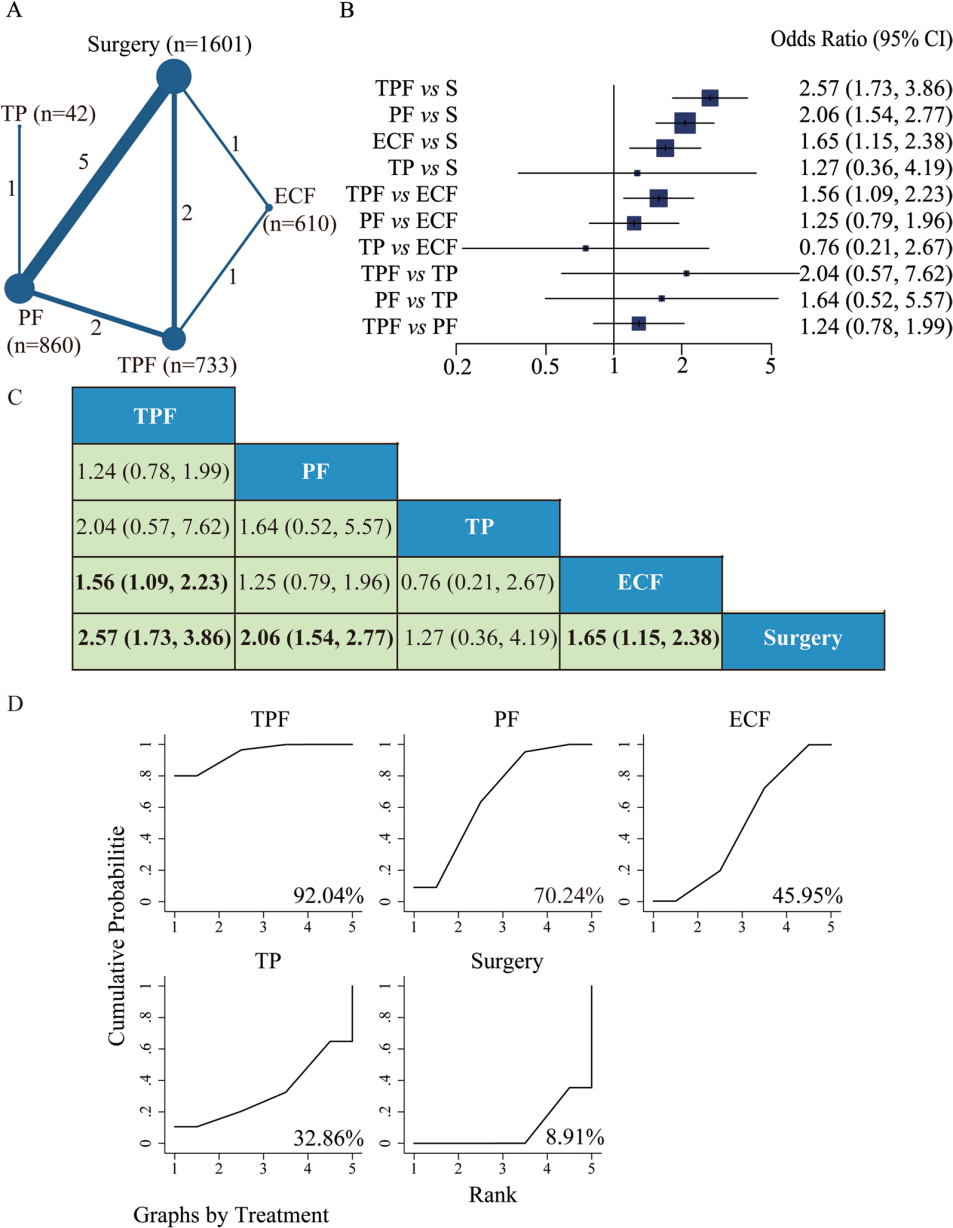
Network meta-analysis of R0 resection rate. **A** Network of R0 resection rate. The size of the nodes and the thickness of the edges are weighted according to the number of studies evaluating each treatment and direct comparison, respectively. **B** Forest plots of comparisons for R0 resection rate (S, surgery). **C** Comparative effectiveness of neoadjuvant treatments in network meta-analysis. Hazard ratio (HR) (95% CI) for comparisons is in cells in common between column-defining and row-defining treatment. Bold cells are significant. HR < 1 favors column-defining treatment. **D** The SUCRA values of each regimen and surgery

R0 resection rate after NAC was reported in all 12 studies. The network plot constituted a closed loop (Fig. 2A). As shown in Fig. 2B, C, the regimens of TPF (OR 2.57, 95% CI 1.73 to 3.86, *P* < 0.001), PF (OR 2.06, 95% CI 1.54 to 2.77, *P* < 0.001), and ECF (OR 1.65, 95% CI 1.15 to 2.38, *P* = 0.007) could improve the R0 resection rate compared with surgery, whereas TP did not enhance R0 resectability. The SUCRA values were 92.04% for TPF regimen, 70.24% for PF regimen, 45.95% for ECF regimen and 32.86% for TP regimen, and the SUCRA value for surgery was 8.91% (Fig. 2D). Importantly, TPF regimen showed significant improvement in curative resection rate *versus* ECF regimen (OR 1.56, 95% CI 1.09 to 2.23, *P* = 0.014), and there was no superiority among the remaining regimens.

### Overall survival and disease-free survival

**Fig. 3:**
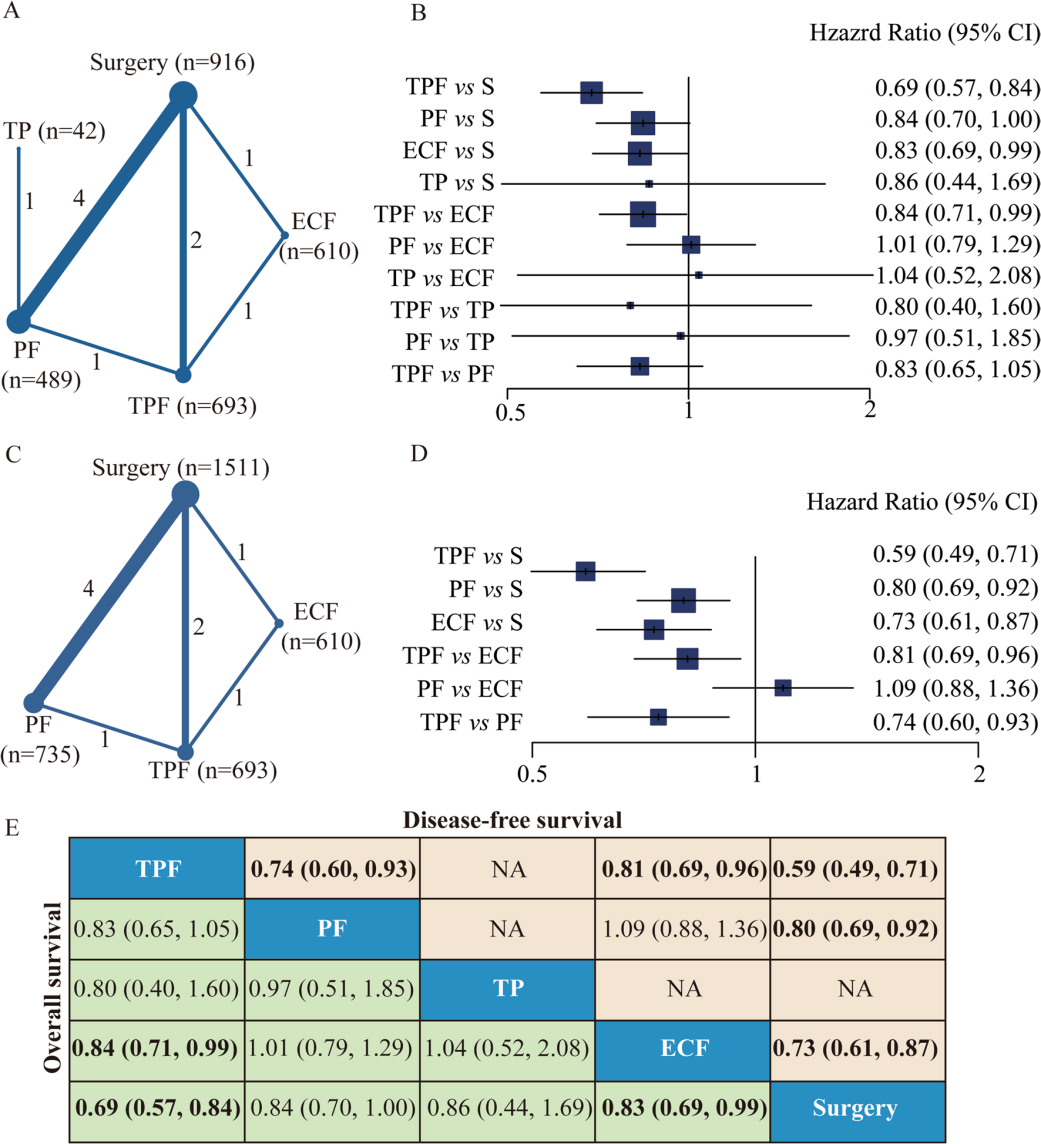
Network meta-analysis of OS and DFS. **A** Network of OS. The size of the nodes and the thickness of the edges are weighted according to the number of studies evaluating each treatment and direct comparison, respectively. **B** Forest plots of comparisons for OS (S, surgery). **C** Network of DFS. The size of the nodes and the thickness of the edges are weighted according to the number of studies evaluating each treatment and direct comparison, respectively. **D** Forest plots of comparisons for DFS (S, surgery). **E** Comparative effectiveness of neoadjuvant treatments in network meta-analysis. Hazard ratio (HR) (95% CI) for comparisons is in cells in common between column-defining and row-defining treatment. Bold cells are significant. For OS, HR  < 1 favors column-defining treatment. For DFS, HR  < 1 favors row-defining treatment

Eleven of 12 studies provided the results of OS and/or DFS. The network plots for OS or DFS constituted a closed loop (Fig. 3A, C). As shown in Fig. 3B, E, the triplet regimens including TPF (HR 0.69, 95% CI 0.57 to 0.84, *P* < 0.001) and ECF (HR 0.83, 95% CI 0.69 to 0.99, *P* = 0.043) could improve OS of the patients *versus* surgery, while the doublet regimens, PF and TP, did not have significant OS benefit. The SUCRA values were 91.35% for TPF regimen, 52.26% for ECF regimen, 50.84% for PF regimen and 46.13% for TP regimen. The SUCRA value for surgery was 9.42%. The pairwise comparison indicated that TPF regimen significantly improves OS *versus* ECF regimen (HR 0.84, 95% CI 0.71 to 0.99, *P* = 0.039), and there was no superiority among the remaining regimens.

As shown in Fig. 3D, E, the regimens of TPF (HR 0.59, 95% CI 0.49 to 0.71, *P* < 0.001), ECF (HR 0.73, 95% CI 0.61 to 0.87, *P* < 0.001), and PF (HR 0.80, 95% CI 0.69 to 0.92, *P* = 0.002) could improve DFS of the patients *versus* surgery, and the study of TP regime did not provide the result of DFS. The SUCRA values were 99.64% for TPF regimen, 59.96% for ECF regimen and 40.37% for PF regimen, and the SUCRA value for surgery was less than 1.0%. Importantly, TPF regimen had superiority in DFS over PF regimen (HR 0.74, 95% CI 0.60 to 0.93, *P* = 0.007) and ECF regimen (HR 0.81, 95% CI 0.69 to 0.96, *P* = 0.012), respectively. There was no significant difference in DFS between ECF regimen and PF regimen.

### Pathological complete response

**Fig. 4:**
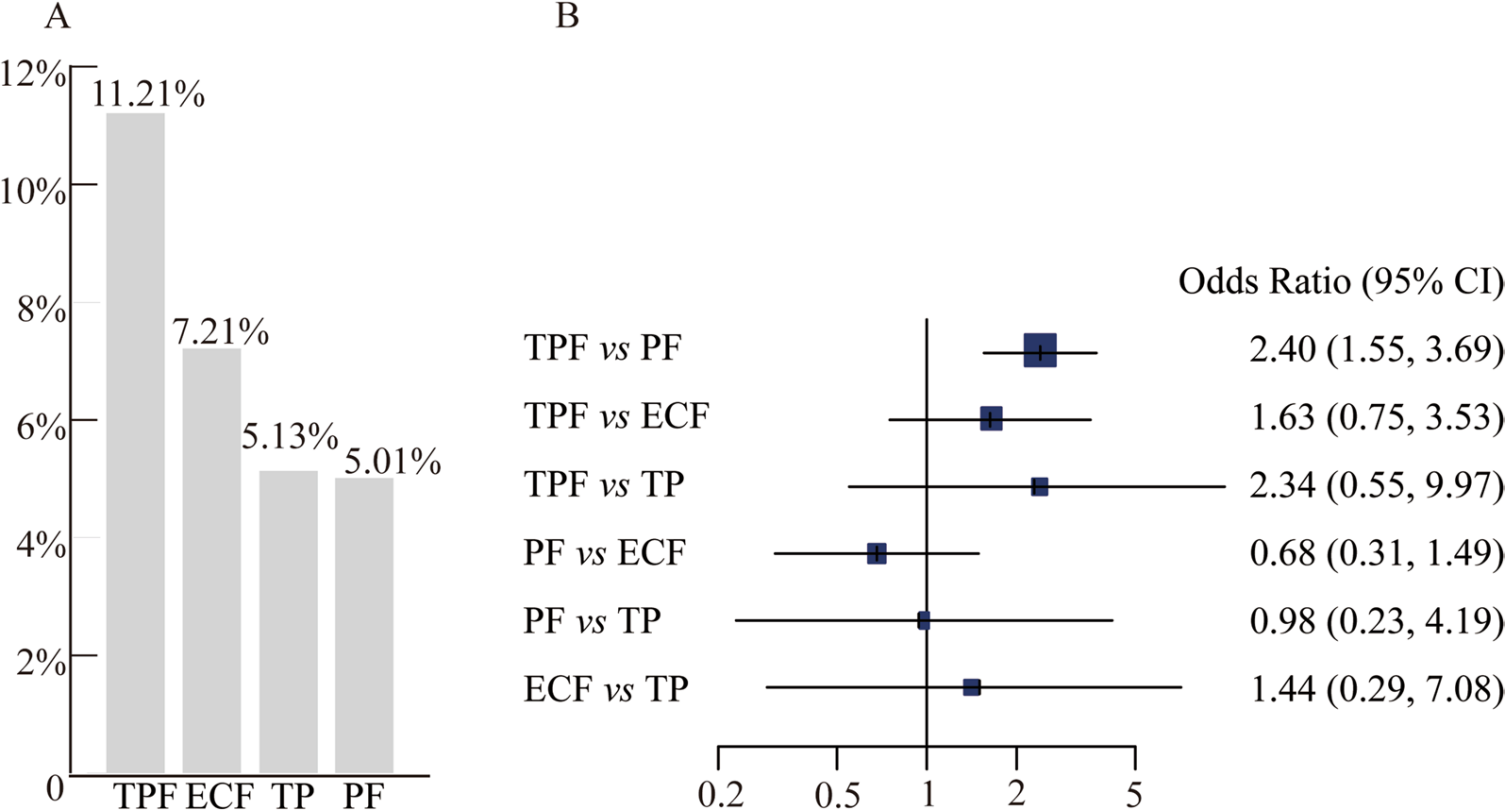
The pCR rate of each regimen. **A** The pCR rate was 11.21% for TPF regimen, 7.21% for ECF regimen, 5.01% for PF regimen and 5.13% for TP regimen. **B** Forest plots of comparisons for pathological complete response rate

Postoperative pathological remission was analyzed in 11 of 12 studies. The patient number with pCR in each study was collected, and the pCR rate of each regimen was calculated. As shown in Fig. 4A, the pCR rate was 11.21% for TPF regimen, 7.21% for ECF regimen, 5.01% for PF regimen, and 5.13% for TP regimen. Importantly, the TPF regimen showed significant improvement in pCR compared with PF regimen (OR 2.40, 95% CI 1.55 to 3.69, *P* < 0.001), and there was no superiority among the remaining regimens (Fig. 4B).

### Safety assessment

Nine of 12 studies reported the NAC-related complications. The grade 3/4 adverse events and postoperative 30-day mortality were reviewed systematically. The regimens of TPF, ECF, and PF showed certain incidences of adverse events and postoperative 30-day mortality (Additional file 1: Figure S1A). The study of TP regimen did not provide the results of adverse events.

As for hematological events, TPF and ECF regimens had higher incidence of neutropenia and leucopenia than PF regimen. The TPF regimen had more leucopenia than ECF regimen. The ECF regimen had higher incidence of serious thrombocytopenia. As for gastroenterological events, the highest incidence of nausea and vomiting occurred in ECF regimen. It was also higher in TPF regimen than that in PF regimen. The severest diarrhea occurred in TPF regimen, and the incidence of diarrhea was higher in ECF regimen than that in PF regimen. The incidence of fatigue was similar to that of nausea and vomiting among these regimens. In addition, these NAC regimens did not significantly increase the deaths within 30 days after surgery. However, the postoperative 30-day mortality in ECF regimen was increased compared with TPF regimen (RR 3.38, 95% CI 1.39 to 8.21, *P* = 0.007) or PF regimen (RR 2.59, 95% CI 1.33 to 5.05, *P* = 0.005) (Additional file 1: Figure S1B).

### Quality assessment and publication bias

According to the Cochrane risk-of-bias tool, the bias assessment for eligible RCTs is shown in Additional file 2: Figure S2A with no severe risk of bias. The GRADE results of R0 resection rate, OS, and DFS are shown in Additional file 5: Table S3. The result of the comparison-adjusted funnel plots for R0 resection rate (Additional file 2: Figure S2B) did not show evidence of obvious asymmetry, suggesting the absence of publication bias.

## Discussion

To our knowledge, this is the first network meta-analysis of RCTs to explore the effectiveness and safety of the NAC regimens for LAGC. The results indicate that the regimens of TPF, PF, and ECF enhance R0 resection rate and improve pCR, which results in survival benefits for the LAGC patients. Importantly, the taxane-based triplet regimen of TPF has been demonstrated to have significant advantages of R0 resectability, OS, DFS, and pCR among these regimens although it shows relatively higher prevalence of grade 3/4 adverse events, which makes TPF the optimal regimen for LAGC patients with good performance status.

The primary objective of NAC for LAGC is to increase R0 resection rate and pCR [8]. In this analysis, the regimens of TPF, PF, and ECF could significantly improve R0 resectability. Importantly, the effects of NAC regimens on R0 resection rate are consistent with those on survivals. Even if some studies suggested that the increased R0 resection rate usually cannot translate into a survival benefit [9], our study promotes us to attribute the survival advantages of NAC to the improved curative resectability. Basi et al. further suggested that R0 resection status can effectively predict mid-term survival in LAGC patients [41]. Intriguingly, TPF regimen had the superiority of R0 resectability over ECF regimen, and no difference was observed among TPF, PF, and TP regimen. Two grading systems, Becker or Mandard, are accepted to assess the tumor regression following NAC for LAGC [23]. Although they have different rating criterion, pCR remains consistent between them. In this analysis, the proportion of patients who achieved pCR ranged from 5.01% to 11.21%. Importantly, TPF regimen showed meaningful improvement in pCR compared with PF regimen, which is consistent with the increased R0 resectability and survival benefits. Major pathologic response has been demonstrated to be associated with the improvement of survival in LAGC patients [7, 42]. The study by Kurokawa et al. has indicated that pathological response exhibits higher response assessment validity and yields the best surrogate endpoint for OS [43].

The goal of any treatment strategy for cancer is to improve survival. Several meta-analyses have demonstrated the clinically significant survival benefits of NAC for LAGC patients compared with the upfront surgery or surgery *plus* postoperative adjuvant chemotherapy [14, 44, 45], and NAC even brings more survival benefits than postoperative chemotherapy for resectable gastric and GEJ cancer [46]. In this network meta-analysis, the triplet chemoagents, TPF and ECF, showed significant benefits of OS and DFS *versus* surgery. However, PF regimen exhibited only DFS benefit without OS benefit, and TP regimen had no survival benefit. This is an extraordinary finding. Although the study of Ychou et al. demonstrated perioperative chemotherapy using fluorouracil*/*cisplatin significantly increase DFS and OS in LAGC patients [17], several other trials did not show OS benefits of PF regimen [9, 22, 29]. Zhang et al. has reported that perioperative S-1/oxaliplatin (SOX) shows a clinically meaningful improvement of 3-year DFS compared with adjuvant capecitabine/oxaliplatin (CapOx) in patients with D2 gastrectomy [31]. SOX regimen has been recommended as NAC for potentially curable gastric cancer in CSCO guidelines [12]. The results of OS in Zhang’s study are worthy to be awaited to approve this recommendation. Importantly, TPF regimen showed significant advantages in OS and DFS over ECF regimen, and had DFS superiority over PF regimen, suggesting the optimality of TPF as NAC regimen for LAGC.

According to ethical principles, NAC is usually followed by postoperative adjuvant chemotherapy in LAGC patients, namely perioperative chemotherapy. Among these enrolled studies, patients in the intervention groups of two early studies received NAC without postoperative adjuvant chemotherapy [9, 18], and patients in the control groups of three early studies only underwent radical surgery without any chemotherapy [4, 9, 17]. Our analysis aimed to elucidate the NAC-related survival benefits for LAGC patients, but the subsequent postoperative adjuvant chemotherapy may influence the survivals. A latest meta-analysis demonstrated that perioperative triplet-based chemotherapy improves both OS and DFS compared to surgery alone or other preoperative strategies for gastric and GEJ cancer [47]. Currently, we cannot draw conclusion on the survival benefits of different combinations of NAC with postoperative adjuvant chemotherapy from these enrolled studies. Thus, further associated clinical trials are needed.

NAC seems to be associated with increased morbidity and mortality [48]. The safety assessment indicated that the triplet regimens had higher incidence of grade 3/4 adverse events, especially neutropenia, leucopenia, nausea and vomiting and fatigue, than the doublet therapy. Importantly, there were more leucopenia and diarrhea in TPF regimen than those in ECF regimen, and more nausea and vomiting and fatigue in ECF regimen than those in TPF regimen. Furthermore, the deaths within 30 days after surgery in ECF regimen were much more than those in TPF regimen and PF regimen. These results indicate that TPF regimen at least has the similar safety as ECF regimen.

Our study has some limitations. Although TPF regimen was shown to be the optimal option, we cannot perform further network meta-analysis for the optimal specific regimen in the category of TPF such as FLOT due to the lack of relevant studies. In addition, the optimal number of cycles is also an important issue for optimal regimen. However, we cannot draw clear conclusion of treatment duration of TPF regimen. The duration of these eligible RCTs ranged from 2 to 4 cycles, and the stratified analysis showed that the 3 or 4 cycles of NAC had more survival benefits than 2 cycles (Additional file 6: Table S4). Several clinical trials have been conducted for the optimal duration of certain regimens for LAGC [20, 21, 25, 26, 48]. An ongoing randomized controlled trial, RESONANCE-II [49], which evaluates the efficacy and safety of three *versus* six cycles of NAC SOX for LAGC patients, deserves to be expected.

## Conclusions

The individualized selection of chemoagents is difficult. This study identified the triplet regimen of TPF to be the optimal NAC regimen for LAGC. Certainly, further clinical trials are warranted to ascertain the efficacy of taxane-based triplet regimen and optimal cycles of treatment.

## Supplementary Information

**Additional file 1: Figure S1.** Systematic review of NAC-related grade 3/4 adverse events and postoperative 30-day mortality. (A) The frequency of grade 3/4 adverse events and postoperative 30-day mortality in each regimen. (B) Forest plots of comparisons for grade 3/4 adverse events and postoperative 30-day mortality (S, surgery).

**Additional file 2: Figure S2.** Quality assessment and publication bias. (A) Risk of bias graph for all studies included. (B) The comparison-adjusted funnel plots for R0 resection rate.

**Additional file 3: Table S1.** The MeSH terms and the related entry terms.

**Additional file 4: Table S2.** Heterogeneity analysis and node-splitting analysis of inconsistency.

**Additional file 5: Table S3.** Estimates of effects and quality ratings for comparison of regimens of neoadjuvant chemotherapy for locally advanced gastric cancer.

**Additional file 6: Table S4.** The stratified analysis of OS, DFS and R0 resectability according to treatment duration.

## Data Availability

The datasets used and/or analyzed during the current study are available from the corresponding author upon reasonable request.

## Abbreviations

NAC: Neoadjuvant chemotherapy
OR: Odds ratio
RR: Risk ratio
HR: Hazard ratio
CI: Confidence interval
pCR: Pathological complete response
OS: Overall survival
DFS: Disease-free survival
TPF: Taxane and platinum plus fluoropyrimidine
ECF: Epirubicin and cisplatin plus fluorouracil
PF: Platinum plus fluoropyrimidine
TP: Taxane plus platinum
GEJ: Gastroesophageal junction
AC: Adjuvant chemotherapy
LAGC: Localized advanced gastric and GEJ cancer
ESMO: European Society for Medical Oncology
JGCA: Japanese Gastric Cancer Association
CSCO: Chinese Society of Clinical Oncology
RCTs: Randomized controlled trials
AEs: Adverse events
PRISMA-NMA: Preferred Reporting Items for Systematic Reviews and Meta-Analyses extension statement for network meta-analyses
SE: Standard error
GRADE: Grading of Recommendations Assessment, Development and Evaluation
SUCRA: Surface under the cumulative ranking curve
SOX: S-1/oxaliplatin
CapOx: Capecitabine/oxaliplatin
