# Supplementary figures and images for: The optimal neoadjuvant chemotherapy regimen for locally advanced gastric and gastroesophageal junction adenocarcinoma: a systematic review and Bayesian network meta-analysis
Source: Eur J Med Res. 2022 Nov 9;27:239. doi: 10.1186/s40001-022-00878-7 (PMC9648003; doi:10.1186/s40001-022-00878-7)

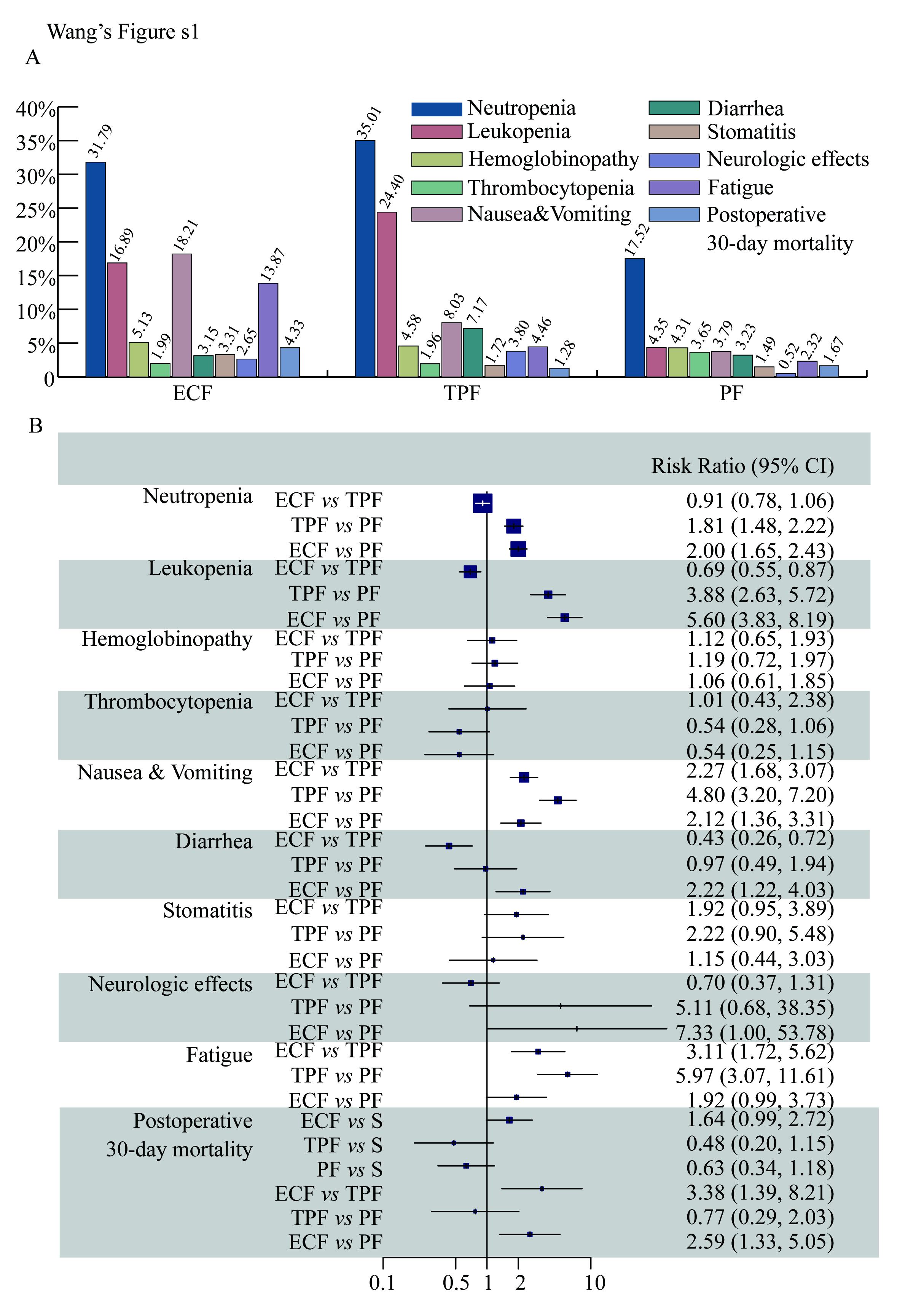

Supplement: Supplementary file 1 — Additional file 1: Figure S1. Systematic review of NAC-related grade 3/4 adverse events and postoperative 30-day mortality. (A) The frequency of grade 3/4 adverse events and postoperative 30-day mortality in each regimen. (B) Forest plots of comparisons for grade 3/4 adverse events and postoperative 30-day mortality (S, surgery). [file 40001_2022_878_MOESM1_ESM.tif]

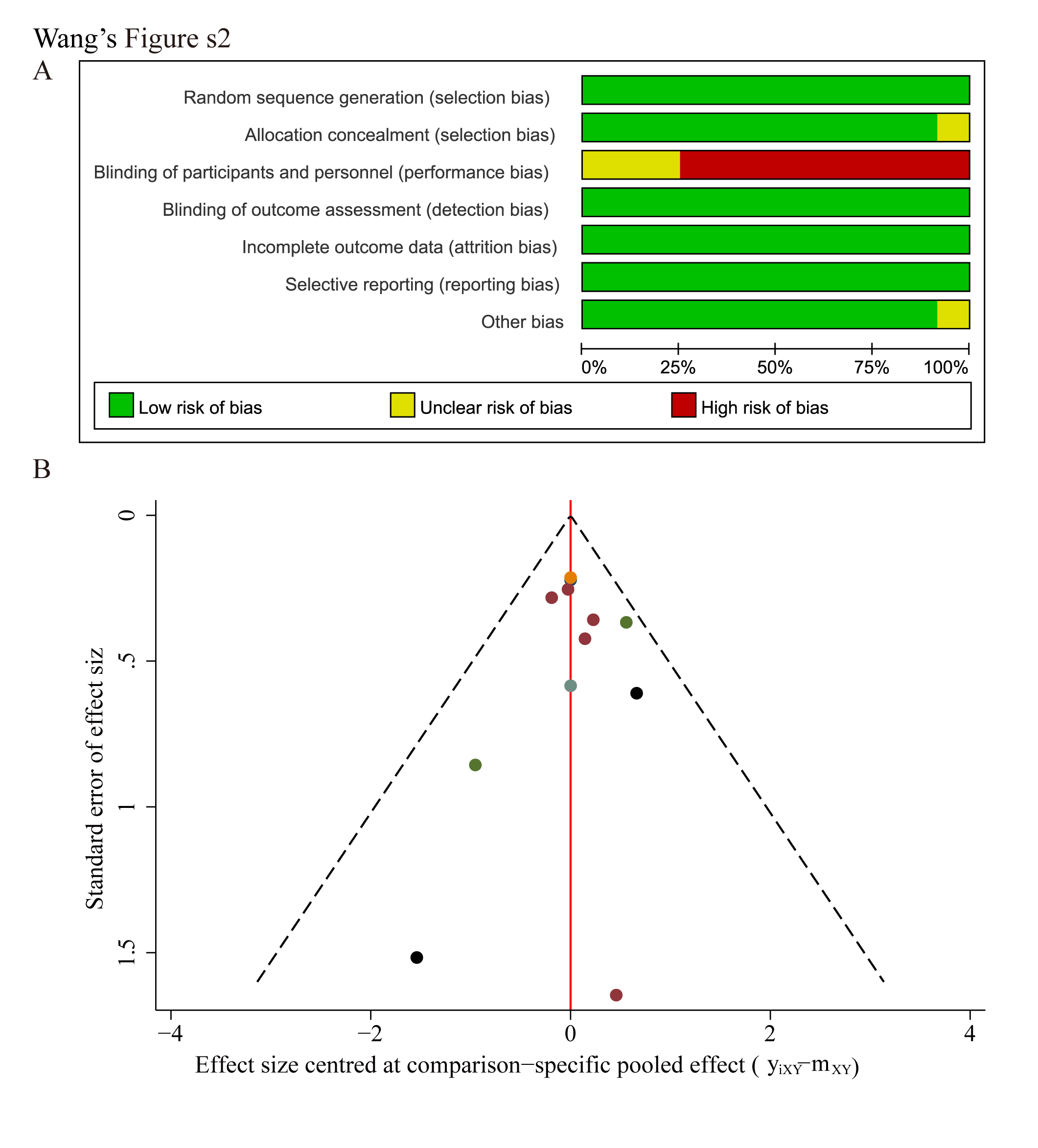

Supplement: Supplementary file 2 — Additional file 2: Figure S2. Quality assessment and publication bias. (A) Risk of bias graph for all studies included. (B) The comparison-adjusted funnel plots for R0 resection rate. [file 40001_2022_878_MOESM2_ESM.tif]
