# Supplementary material for: The optimal neoadjuvant chemotherapy regimen for locally advanced gastric and gastroesophageal junction adenocarcinoma: a systematic review and Bayesian network meta-analysis
Source: Eur J Med Res. 2022 Nov 9;27:239. doi: 10.1186/s40001-022-00878-7 (PMC9648003; doi:10.1186/s40001-022-00878-7)
Supplement: Supplementary file 3 — Additional file 3: Table S1. The MeSH terms and the related entry terms. [file 40001_2022_878_MOESM3_ESM.docx]

**Table s1. The MeSH terms and the related entry terms**

**MeSH term**: Stomach Neoplasms

Esophagogastric Junction

**Entry terms**:

Neoplasm, Stomach

Stomach Neoplasm

Neoplasms, Stomach

Gastric Neoplasms

Gastric Neoplasm

Neoplasm, Gastric

Neoplasms, Gastric

Cancer of Stomach

Stomach Cancers

Gastric Cancer

Cancer, Gastric

Cancers, Gastric

Gastric Cancers

Stomach Cancer

Cancer, Stomach

Cancers, Stomach

Cancer of the Stomach

Gastric Cancer, Familial Diffuse

**MeSH term**: Neoadjuvant Therapy

**Entry terms**:

Neoadjuvant Therapies

Therapy, Neoadjuvant

Neoadjuvant Treatment

Neoadjuvant Treatments

Treatment, Neoadjuvant

Neoadjuvant Systemic Therapy

Neoadjuvant Systemic Therapies

Systemic Therapy, Neoadjuvant

Therapy, Neoadjuvant Systemic

Neoadjuvant Systemic Treatment

Neoadjuvant Systemic Treatments

Systemic Treatment, Neoadjuvant

Treatment, Neoadjuvant Systemic

Neoadjuvant Chemotherapy

Chemotherapy, Neoadjuvant

Neoadjuvant Chemotherapies

Neoadjuvant Chemotherapy Treatment

Chemotherapy Treatment, Neoadjuvant

Neoadjuvant Chemotherapy Treatments

Treatment, Neoadjuvant Chemotherapy

**MeSH term**: randomized controlled trial

**Entry terms**:

randomized

placebo

RCT

**PubMed**

(((("Stomach Neoplasms"[Mesh])) OR ("Esophagogastric Junction"[Mesh]) OR ((Neoplasm, Stomach[Title/Abstract]) OR (Stomach Neoplasm[Title/Abstract]) OR (Neoplasms, Stomach[Title/Abstract]) OR (Gastric Neoplasms[Title/Abstract]) OR (Gastric Neoplasm[Title/Abstract]) OR (Neoplasm, Gastric[Title/Abstract]) OR (Neoplasms, Gastric[Title/Abstract]) OR (Cancer of Stomach[Title/Abstract]) OR (Stomach Cancers[Title/Abstract]) OR (Gastric Cancer[Title/Abstract]) OR (Cancer, Gastric[Title/Abstract]) OR (Cancers, Gastric[Title/Abstract]) OR (Gastric Cancers[Title/Abstract]) OR (Stomach Cancer[Title/Abstract]) OR (Cancer, Stomach[Title/Abstract]) OR (Cancers, Stomach[Title/Abstract]) OR (Cancer of the Stomach[Title/Abstract]) OR (Gastric Cancer, Familial Diffuse[Title/Abstract]))) AND (("Neoadjuvant Therapy"[Mesh]) OR ((Neoadjuvant Therapies[Title/Abstract]) OR (Therapy, Neoadjuvant[Title/Abstract]) OR (Neoadjuvant Treatment[Title/Abstract]) OR (Neoadjuvant Treatments[Title/Abstract]) OR (Treatment, Neoadjuvant[Title/Abstract]) OR (Neoadjuvant Systemic Therapy[Title/Abstract]) OR (Neoadjuvant Systemic Therapies[Title/Abstract]) OR (Systemic Therapy, Neoadjuvant[Title/Abstract]) OR (Therapy, Neoadjuvant Systemic[Title/Abstract]) OR (Neoadjuvant Systemic Treatment[Title/Abstract]) OR (Neoadjuvant Systemic Treatments[Title/Abstract]) OR (Systemic Treatment, Neoadjuvant[Title/Abstract]) OR (Treatment, Neoadjuvant Systemic[Title/Abstract]) OR (Neoadjuvant Chemotherapy[Title/Abstract]) OR (Chemotherapy, Neoadjuvant[Title/Abstract]) OR (Neoadjuvant Chemotherapies[Title/Abstract]) OR (Neoadjuvant Chemotherapy Treatment[Title/Abstract]) OR (Chemotherapy Treatment, Neoadjuvant[Title/Abstract]) OR (Neoadjuvant Chemotherapy Treatments[Title/Abstract]) OR (Treatment, Neoadjuvant Chemotherapy[Title/Abstract])))) AND (randomized controlled trial[Publication Type] OR randomized[Title/Abstract] OR placebo[Title/Abstract])

**Web of Science**

TS=(Stomach Neoplasms OR Neoplasm, Stomach OR Stomach Neoplasm OR Neoplasms, Stomach OR Gastric Neoplasms OR Gastric Neoplasm OR Neoplasm, Gastric OR Neoplasms, Gastric OR Cancer of Stomach OR Stomach Cancers OR Gastric Cancer OR Cancer, Gastric OR Cancers, Gastric OR Gastric Cancers OR Stomach Cancer OR Cancer, Stomach OR Cancers, Stomach OR Cancer of the Stomach OR Gastric Cancer, Familial Diffuse)

TS=(Neoadjuvant Therapy OR Neoadjuvant Therapies OR Therapy, Neoadjuvant OR Neoadjuvant Treatment OR Neoadjuvant Treatments OR Treatment, Neoadjuvant OR Neoadjuvant Systemic Therapy OR Neoadjuvant Systemic Therapies OR Systemic Therapy, Neoadjuvant OR Therapy, Neoadjuvant Systemic OR Neoadjuvant Systemic Treatment OR Neoadjuvant Systemic Treatments OR Systemic Treatment, Neoadjuvant OR Treatment, Neoadjuvant Systemic OR Neoadjuvant Chemotherapy OR Chemotherapy, Neoadjuvant OR Neoadjuvant Chemotherapies OR Neoadjuvant Chemotherapy Treatment OR Chemotherapy Treatment, Neoadjuvant OR Neoadjuvant Chemotherapy Treatments OR Treatment, Neoadjuvant Chemotherapy)

TS=(randomized controlled trial OR randomized OR placebo OR RCT)

**Embase**

('stomach'/exp OR stomach) AND ('neoplasms'/exp OR neoplasms)

'neoplasm, stomach':ab,ti OR 'stomach neoplasm':ab,ti OR 'neoplasms, stomach':ab,ti OR 'gastric neoplasms':ab,ti OR 'gastric neoplasm':ab,ti OR 'neoplasm, gastric':ab,ti OR 'neoplasms, gastric':ab,ti OR 'cancer of stomach':ab,ti OR 'stomach cancers':ab,ti OR 'gastric cancer':ab,ti OR 'cancer, gastric':ab,ti OR 'cancers, gastric':ab,ti OR 'gastric cancers':ab,ti OR 'stomach cancer':ab,ti OR 'cancer, stomach':ab,ti OR 'cancers, stomach':ab,ti OR 'cancer of the stomach':ab,ti OR 'gastric cancer, familial diffuse':ab,ti

neoadjuvant AND therapy

'neoadjuvant therapies':ab,ti OR 'therapy, neoadjuvant':ab,ti OR 'neoadjuvant treatment':ab,ti OR 'neoadjuvant treatments':ab,ti OR 'treatment, neoadjuvant':ab,ti OR 'neoadjuvant systemic therapy':ab,ti OR 'neoadjuvant systemic therapies':ab,ti OR 'systemic therapy, neoadjuvant':ab,ti OR 'therapy, neoadjuvant systemic':ab,ti OR 'neoadjuvant systemic treatment':ab,ti OR 'neoadjuvant systemic treatments':ab,ti OR 'systemic treatment, neoadjuvant':ab,ti OR 'treatment, neoadjuvant systemic':ab,ti OR 'neoadjuvant chemotherapy':ab,ti OR 'chemotherapy, neoadjuvant':ab,ti OR 'neoadjuvant chemotherapies':ab,ti OR 'neoadjuvant chemotherapy treatment':ab,ti OR 'chemotherapy treatment, neoadjuvant':ab,ti OR 'neoadjuvant chemotherapy treatments':ab,ti OR 'treatment, neoadjuvant chemotherapy':ab,ti

'randomized controlled trial':ab,ti OR 'randomized':ab,ti OR 'placebo':ab,ti OR 'rct':ab,ti

**Cochrane**

Stomach Neoplasms

(Neoplasm, Stomach):ab,ti,kw OR (Stomach Neoplasm):ab,ti,kw OR (Neoplasms, Stomach):ab,ti,kw OR (Gastric Neoplasms):ab,ti,kw OR (Gastric Neoplasm):ab,ti,kw OR (Neoplasm, Gastric):ab,ti,kw OR (Neoplasms, Gastric):ab,ti,kw OR (Cancer of Stomach):ab,ti,kw OR (Stomach Cancers):ab,ti,kw OR (Gastric Cancer):ab,ti,kw OR (Cancer, Gastric):ab,ti,kw OR (Cancers, Gastric):ab,ti,kw OR (Gastric Cancers):ab,ti,kw OR (Stomach Cancer):ab,ti,kw OR (Cancer, Stomach):ab,ti,kw OR (Cancers, Stomach):ab,ti,kw OR (Cancer of the Stomach):ab,ti,kw OR (Gastric Cancer, Familial Diffuse):ab,ti,kw

Neoadjuvant Therapy

(Neoadjuvant Therapies):ab,ti,kw OR (Therapy, Neoadjuvant):ab,ti,kw OR (Neoadjuvant Treatment):ab,ti,kw OR (Neoadjuvant Treatments):ab,ti,kw OR (Treatment, Neoadjuvant):ab,ti,kw OR (Neoadjuvant Systemic Therapy):ab,ti,kw OR (Neoadjuvant Systemic Therapies):ab,ti,kw OR (Systemic Therapy, Neoadjuvant):ab,ti,kw OR (Therapy, Neoadjuvant Systemic):ab,ti,kw OR (Neoadjuvant Systemic Treatment):ab,ti,kw OR (Neoadjuvant Systemic Treatments):ab,ti,kw OR (Systemic Treatment, Neoadjuvant):ab,ti,kw OR (Treatment, Neoadjuvant Systemic):ab,ti,kw OR (Neoadjuvant Chemotherapy):ab,ti,kw OR (Chemotherapy, Neoadjuvant):ab,ti,kw OR (Neoadjuvant Chemotherapies):ab,ti,kw OR (Neoadjuvant Chemotherapy Treatment):ab,ti,kw OR (Chemotherapy Treatment, Neoadjuvant):ab,ti,kw OR (Neoadjuvant Chemotherapy Treatments):ab,ti,kw OR (Treatment, Neoadjuvant Chemotherapy):ab,ti,kw

(randomized controlled trial):ab,ti,kw OR (randomized):ab,ti,kw OR (placebo):ab,ti,kw OR (RCT):ab,ti,kw
