# Supplementary material for: The optimal neoadjuvant chemotherapy regimen for locally advanced gastric and gastroesophageal junction adenocarcinoma: a systematic review and Bayesian network meta-analysis
Source: Eur J Med Res. 2022 Nov 9;27:239. doi: 10.1186/s40001-022-00878-7 (PMC9648003; doi:10.1186/s40001-022-00878-7)
Supplement: Supplementary file 4 — Additional file 4: Table S2. Heterogeneity analysis and node-splitting analysis of inconsistency. [file 40001_2022_878_MOESM4_ESM.docx]

**Table s2. Heterogeneity analysis and node-splitting analysis of inconsistency**

**Heterogeneity analysis**

|  | Model | Dbar | pD | DIC | Global I^2^ values |
| --- | --- | --- | --- | --- | --- |
| R0 resectability | Random | 26.36924 | 18.73132 | 45.10056 |  |
|  | Fixed | 27.18311 | 16.35055 | 43.53366 | 15% |
| OS | Random | 8.754713 | 6.124421 | 14.87913 |  |
|  | Fixed | 10.20516 | 3.988601 | 14.19376 | 12% |
| DFS | Random | 8.373385 | 5.626531 | 13.99992 |  |
|  | Fixed | 10.54912 | 3.005435 | 13.55456 | 24% |

**Node-splitting analysis of inconsistency**

|  | | | | |
| --- | --- | --- | --- | --- |
| **R0 resectability** | **comparison** | ***p*. value** | **CrI** |  |
|  | ECF *vs* Surgery | 0.8478 |  | 0% |
|  | direct |  | -0.49 (-0.93, -0.050) |  |
|  | indirect |  | -0.57 (-1.3, 0.11) |  |
|  | network |  | -0.51 (-0.88, -0.14) |  |
|  | ECF *vs* TPF | 0.8480 |  | 0% |
|  | direct |  | 0.42 (-4.3e-05, 0.84) |  |
|  | indirect |  | 0.50 (-0.18, 1.2) |  |
|  | network |  | 0.44 (0.085, 0.79) |  |
|  | PF *vs* Surgery | 0.1850 |  | 0% |
|  | direct |  | -0.67 (-0.98, -0.38) |  |
|  | indirect |  | -1.5 (-2.7, -0.32) |  |
|  | network |  | -0.72 (-1.0, -0.43) |  |
|  | PF *vs* TPF | 0.1810 |  | 23.3% |
|  | direct |  | -0.43 (-1.6, 0.64) |  |
|  | indirect |  | 0.39 (-0.14, 0.92) |  |
|  | network |  | 0.22 (-0.24, 0.70) |  |
|  | Surgery *vs* TPF | 0.2608 |  | 23.4% |
|  | direct |  | 1.2 (0.61, 1.9) |  |
|  | indirect |  | 0.76 (0.22, 1.3) |  |
|  | network |  | 0.95 (0.55, 1.3) |  |

| **OS** | **comparison** | ***p*. value** | | **CrI** | **I^2^** |
| --- | --- | --- | --- | --- | --- |
|  | PF *vs* Surgery | 0.90357 | |  | 0% |
|  | direct |  | | 0.18 (-0.0063, 0.37) |  |
|  | indirect |  | | 0.15 (-0.40, 0.69) |  |
|  | network |  | | 0.18 (-0.00093, 0.36) |  |
|  | PF *vs* TPF | 0.89919 | |  | 0% |
|  | direct |  | | -0.22 (-0.73, 0.29) |  |
|  | indirect |  | | -0.18 (-0.46, 0.098) |  |
|  | network |  | | -0.19 (-0.44, 0.054) |  |
|  | Surgery *vs* TPF | 0.09006 | |  | 31.4% |
|  | direct |  | | -0.18 (-0.47, 0.11) |  |
|  | indirect |  | | -0.52 (-0.78, -0.26) |  |
|  | network |  | | -0.37 (-0.56, -0.18) |  |
|  | ECF *vs* Surgery | 0.10736 | |  | 61.8% |
|  | direct |  | | 0.29(0.073, 0.50) |  |
|  | indirect |  | | -0.029(-0.35,0.29) |  |
|  | network |  | | 0.19 (0.012, 0.37) |  |
|  | ECF *vs* TPF | 0.10563 | |  | 62.5% |
|  | direct |  | | -0.26 (-0.46, -0.064) |  |
|  | indirect |  | | 0.059 (-0.28, 0.40) |  |
|  | network |  | | -0.18 (-0.35, -0.0083) |  |
|  | | |  |  |  |
| **DFS** | **comparison** | ***p*. value** | | **CrI** | **I^2^** |
|  | PF *vs* Surgery | 0.64347 | |  | 0% |
|  | direct |  | | 0.24 (0.091, 0.38) |  |
|  | indirect |  | | 0.11 (-0.42, 0.64) |  |
|  | network |  | | 0.23 (0.088, 0.37) |  |
|  | PF *vs* TPF | 0.64392 | |  | 0% |
|  | direct |  | | -0.40 (-0.89, 0.094) |  |
|  | indirect |  | | -0.27 (-0.52, -0.027) |  |
|  | network |  | | -0.30 (-0.51, -0.078) |  |
|  | Surgery *vs* TPF | 0.05878 | |  | 45.3% |
|  | direct |  | | -0.33 (-0.60, -0.062) |  |
|  | indirect |  | | -0.69 (-0.94, -0.44) |  |
|  | network |  | | -0.52 (-0.71, -0.34) |  |
|  | ECF *vs* Surgery | 0.11134 | |  | 61.9% |
|  | direct |  | | 0.41 (0.20, 0.63) |  |
|  | indirect |  | | 0.11 (-0.19, 0.42) |  |
|  | network |  | | 0.32 (0.14, 0.49) |  |
|  | ECF *vs* TPF | 0.11047 | |  | 60.9% |
|  | direct |  | | -0.29 (-0.48, -0.095) |  |
|  | indirect |  | | 0.017 (-0.30, 0.34) |  |
|  | network |  | | -0.21 (-0.37, -0.043) |  |
|  | | | | | |
