# Supplementary material for: The optimal neoadjuvant chemotherapy regimen for locally advanced gastric and gastroesophageal junction adenocarcinoma: a systematic review and Bayesian network meta-analysis
Source: Eur J Med Res. 2022 Nov 9;27:239. doi: 10.1186/s40001-022-00878-7 (PMC9648003; doi:10.1186/s40001-022-00878-7)
Supplement: Supplementary file 5 — Additional file 5: Table S3. Estimates of effects and quality ratings for comparison of regimens of neoadjuvant chemotherapy for locally advanced gastric cancer. [file 40001_2022_878_MOESM5_ESM.docx]

**Table s3. Estimates of effects and quality ratings for comparison of regimens of neoadjuvant chemotherapy for locally advanced gastric cancer.**

| **R0 resectability** | OR  (95% CI) | Quality of evidence | OR  (95% CI) | Quality of evidence | OR  (95% CI) | Quality of evidence |
| --- | --- | --- | --- | --- | --- | --- |
| TPF *vs* Surgery | 3.40  (1.80, 6.80) | Moderate  ⊕⊕⊕O | 2.20  (1.30, 3.60) | Moderate  ⊕⊕⊕O | 2.60  (1.70, 3.90) | Moderate  ⊕⊕⊕O |
| TPF *vs* PF | 0.65  (0.21, 1.90) | Moderate  ⊕⊕⊕O | 1.40  (0.86, 2.40) | Low  ⊕⊕OO | 1.20  (0.78, 2.00) | Moderate  ⊕⊕⊕O |
| TPF *vs* ECF | 1.50  (1.00, 2.30) | Moderate  ⊕⊕⊕O | 1.60  (0.83, 3.30) | Low  ⊕⊕OO | 1.60  (1.10, 2.20) | Low  ⊕⊕OO |
| TPF *vs* TP | - | - | 2.10  (0.60, 7.66) | Low  ⊕⊕OO | 2.10  (0.60, 7.66) | Low  ⊕⊕OO |
| Surgery *vs* ECF | 0.62  (0.40, 0.95) | Moderate  ⊕⊕⊕O | 0.57  (0.29, 1.10) | Moderate  ⊕⊕⊕O | 0.60  (0.42, 0.87) | Moderate  ⊕⊕⊕O |
| Surgery *vs* PF | 0.51  (0.38, 0.69) | Moderate  ⊕⊕⊕O | 0.19  (0.05, 0.66) | Moderate  ⊕⊕⊕O | 0.48  (0.36, 0.65) | Moderate  ⊕⊕⊕O |
| Surgery *vs* TP | - | - | 0.79  (0.24, 2.73) | Low  ⊕⊕OO | 0.79  (0.24, 2.73) | Low  ⊕⊕OO |
| TP *vs* ECF | - | - | 0.75  (0.21, 2.59) | Low  ⊕⊕OO | 0.75  (0.21, 2.59) | Low  ⊕⊕OO |
| TP *vs* PF | 0.61  (0.18, 1.90) | Low  ⊕⊕OO | - | - | 0.61  (0.18, 1.90) | Low  ⊕⊕OO |
| ECF *vs* PF | - | - | 0.81  (0.52, 1.28) | Moderate  ⊕⊕⊕O | 0.81  (0.52, 1.28) | Moderate  ⊕⊕⊕O |
| Ratings  *High quality* (⊕⊕⊕⊕)*—*We are very confident that the true effect lies close to that of the estimate of the effect.  *Moderate quality* (⊕⊕⊕O)*—*We are moderately confident in the effect estimate: the true effect is likely to be close to the estimate of the effect, but there is a possibility that it is substantially different.  *Low quality* (⊕⊕OO)—Our confidence in the effect estimate is limited: the true effect may be substantially different from the estimate of the effect.  *Very low quality* (⊕OOO)—We have very little confidence in the effect estimate: the true effect is likely to be substantially different from the estimate of effect. | | | | | | |

| Comparison | Direct evidence | | Indirect evidence | | Network meta-analysis | |
| --- | --- | --- | --- | --- | --- | --- |
| **Overall Survival** | HR (95%CI) | Quality of evidence | HR  (95%CI) | Quality of evidence | HR  (95%CI) | Quality of evidence |
| TPF *vs* Surgery | 0.83  (0.62, 1.10) | Moderate  ⊕⊕⊕O | 0.60  (0.46, 0.77) | Moderate  ⊕⊕⊕O | 0.69  (0.57, 0.84) | Moderate  ⊕⊕⊕O |
| TPF *vs* PF | 0.80  (0.48, 1.30) | Moderate  ⊕⊕⊕O | 0.83  (0.63, 1.10) | Moderate  ⊕⊕⊕O | 0.83  (0.65, 1.05) | Moderate  ⊕⊕⊕O |
| TPF *vs* ECF | 0.77  (0.63, 0.94) | Moderate  ⊕⊕⊕O | 1.10  (0.76, 1.50) | Moderate  ⊕⊕⊕O | 0.84  (0.71, 0.99) | Moderate  ⊕⊕⊕O |
| TPF *vs* TP | - | - | 0.80  (0.40, 1.60) | Low  ⊕⊕OO | 0.80  (0.40, 1.60) | Low  ⊕⊕OO |
| Surgery *vs* ECF | 1.30  (1.10, 1.70) | Moderate  ⊕⊕⊕O | 0.97  (0.70, 1.30) | Low  ⊕⊕OO | 1.21  (1.01, 1.45) | Low  ⊕⊕OO |
| Surgery *vs* PF | 1.20  (0.99, 1.40) | Moderate  ⊕⊕⊕O | 1.20  (0.67, 2.00) | Low  ⊕⊕OO | 1.20  (1.00, 1.43) | Moderate  ⊕⊕⊕O |
| Surgery *vs* TP | - | - | 1.16  (0.59, 2.26) | Low  ⊕⊕OO | 1.16  (0.59, 2.26) | Low  ⊕⊕OO |
| TP *vs* ECF | - | - | 1.04  (0.52, 2.08) | Low  ⊕⊕OO | 1.04  (0.52, 2.08) | Low  ⊕⊕OO |
| TP *vs* PF | 1.03  (0.54, 1.97) | Low  ⊕⊕OO | - | - | 1.03  (0.54, 1.97) | Low |
| ECF *vs* PF | - | - | 0.99  (0.77, 1.26) | Moderate  ⊕⊕⊕O | 0.99  (0.77,1.26) | Moderate  ⊕⊕⊕O |
| Ratings  *High quality* (⊕⊕⊕⊕)*—*We are very confident that the true effect lies close to that of the estimate of the effect.  *Moderate quality* (⊕⊕⊕O)*—*We are moderately confident in the effect estimate: the true effect is likely to be close to the estimate of the effect, but there is a possibility that it is substantially different.  *Low quality* (⊕⊕OO)—Our confidence in the effect estimate is limited: the true effect may be substantially different from the estimate of the effect.  *Very low quality* (⊕OOO)—We have very little confidence in the effect estimate: the true effect is likely to be substantially different from the estimate of effect. | | | | | | |
| **Disease-free Survival** | HR  (95% CI) | Quality of evidence | HR  (95% CI) | Quality of evidence | HR  (95% CI) | Quality of evidence |
| TPF *vs* Surgery | 0.72  (0.55, 0.94) | Moderate  ⊕⊕⊕O | 0.50  (0.39, 0.65) | Low  ⊕OOO | 0.59  (0.49, 0.71) | Moderate  ⊕⊕⊕O |
| TPF *vs* PF | 0.67  (0.41, 1.10) | Moderate  ⊕⊕⊕O | 0.76  (0.60, 0.98) | Low  ⊕OOO | 0.74  (0.60, 0.93) | Moderate  ⊕⊕⊕O |
| TPF *vs* ECF | 0.75  (0.62, 0.91) | Moderate⊕⊕⊕O | 1.00  (0.74, 1.40) | Low  ⊕OOO | 0.81  (0.69, 0.96) | Moderate  ⊕⊕⊕O |
| Surgery *vs* ECF | 1.50  (1.20, 1.90) | Low  ⊕OOO | 1.10  (0.82, 1.50) | Moderate  ⊕⊕⊕O | 1.37  (1.15, 1.64) | Moderate  ⊕⊕⊕O |
| Surgery *vs* PF | 1.30  (1.10, 1.50) | Moderate  ⊕⊕⊕O | 1.10  (0.65, 1.90) | Moderate  ⊕⊕⊕O | 1.25  (1.09, 1.44) | Moderate  ⊕⊕⊕O |
| ECF *vs* PF | - | - | 0.91  (0.74, 1.14) | Low  ⊕OOO | 0.91  (0.74, 1.14) | Low  ⊕OOO |
| Ratings  *High quality* (⊕⊕⊕⊕)*—*We are very confident that the true effect lies close to that of the estimate of the effect.  *Moderate quality* (⊕⊕⊕O)*—*We are moderately confident in the effect estimate: the true effect is likely to be close to the estimate of the effect, but there is a possibility that it is substantially different.  *Low quality* (⊕⊕OO)—Our confidence in the effect estimate is limited: the true effect may be substantially different from the estimate of the effect.  *Very low quality* (⊕OOO)—We have very little confidence in the effect estimate: the true effect is likely to be substantially different from the estimate of effect. | | | | | | |
