# Supplementary material for: The optimal neoadjuvant chemotherapy regimen for locally advanced gastric and gastroesophageal junction adenocarcinoma: a systematic review and Bayesian network meta-analysis
Source: Eur J Med Res. 2022 Nov 9;27:239. doi: 10.1186/s40001-022-00878-7 (PMC9648003; doi:10.1186/s40001-022-00878-7)
Supplement: Supplementary file 6 — Additional file 6: Table S4. The stratified analysis of OS, DFS and R0 resectability according to treatment duration. [file 40001_2022_878_MOESM6_ESM.docx]

**Table s4. The stratified analysis of OS, DFS and R0 resectability according to treatment duration**

| Comparisons | Overall survival  HR (95% CI) | Disease-free survival  HR (95% CI) | R0 resectability  OR (95% CI) |
| --- | --- | --- | --- |
| **2 cycles** |  | | |
| TPF *vs* Surgery | NA | NA | 1.39 (0.23, 7.77) |
| PF *vs* Surgery | 0.93 (0.73, 1.18) | 0.91 (0.72, 1.15) | **1.82 (1.16, 2.90)** |
| TP *vs* Surgery | NA | NA | 0.70 (0.12, 3.58) |
| TPF *vs* PF | NA | NA | 0.76 (0.13, 4.01) |
| TPF *vs* TP | NA | NA | 1.99 (0.19, 21.55) |
| PF *vs* TP | NA | NA | 2.58 (0.55, 14.73) |
| **3~4 cycles** |  | | |
| TPF *vs* Surgery | **0.69 (0.57, 0.85)** | **0.60 (0.50, 0.73)** | **2.68 (1.77, 4.10)** |
| PF *vs* Surgery | **0.69 (0.50, 0.95)** | **0.73 (0.60, 0.88)** | **2.22 (1.51, 3.32)** |
| TP *vs* Surgery | NA | NA | 2.37 (0.33, 16.47) |
| ECF *vs* Surgery | 0.83 (0.69, 1.00) | **0.74 (0.61, 0.88)** | **1.69 (1.17, 2.48)** |
| TPF *vs* PF | 1.00 (0.69, 1.47) | 0.83 (0.64, 1.09) | 1.21 (0.70, 2.11) |
| TPF *vs* ECF | 0.84 (0.71, 1.00) | **0.82 (0.69, 0.97)** | **1.58 (1.10, 2.30)** |
| TPF *vs* TP | NA | NA | 1.13 (0.16, 8.37) |
| PF *vs* ECF | 0.83 (0.58, 1.20) | 0.99 (0.76, 1.28) | 1.31 (0.77, 2.24) |
| PF *vs* TP | NA | NA | 0.93 (0.14, 6.40) |
| TP *vs* ECF | NA | NA | 1.40 (0.19, 10.08) |
